# Management of allergic rhinitis symptoms in the pharmacy Pocket guide 2022

**DOI:** 10.1002/clt2.12183

**Published:** 2022-10-05

**Authors:** Olga Lourenço, Biljana Cvetkovski, Vicky Kritikos, Rachel House, Sophie Scheire, Elisio M Costa, João A. Fonseca, Enrica Menditto, Anna Bedbrook, Slawomir Bialek, Vitalis Briedis, Koen Boussery, G. Walter Canonica, Tari Haahtela, Piotr Kuna, Joaquim Mullol, Valentina Orlando, Boleslaw Samolinski, Dana Wallace, Catherine Duggan, Ema Paulino, Gonçalo S. Pinto, Lars‐Åke Söderlund, Jean Bousquet, Sinthia Bosnic‐Anticevich

**Affiliations:** ^1^ Faculty of Health Sciences and CICS – UBI Health Sciences Research Centre University of Beira Interior Covilhã Portugal; ^2^ Woolcock Institute of Medical Research and University of Sydney Glebe NSW Australia; ^3^ Sydney Local Health District Sydney NSW Australia; ^4^ Pharmaceutical Care Unit Faculty of Pharmaceutical Sciences Ghent University Ghent Belgium; ^5^ UCIBIO, REQUINTE Faculty of Pharmacy and Competence Center on Active and Healthy Ageing of University of Porto (Porto4Ageing) Porto Portugal; ^6^ MEDCIDS ‐ Department of Community Medicine Information and Health Decision Sciences Faculty of Medicine CINTESIS – Center for Health Technology and Services Research RISE – Health Research Network University of Porto Porto Portugal; ^7^ Department of Pharmacy CIRFF Center of Pharmacoeconomics and Drug Utilization Research University of Naples Federico II Naples Italy; ^8^ ARIA Montpellier France; ^9^ MASK‐air Montpellier France; ^10^ Department of Biochemistry and Clinical Chemistry Faculty of Pharmacy Medical University of Warsaw Warsaw Poland; ^11^ Department of Clinical Pharmacy of Lithuanian University of Health Sciences Kaunas Lithuania; ^12^ Department of Biomedical Sciences Humanitas University Pieve Emanuele, Italy & Personalized Medicine, Asthma and Allergy Humanitas Clinical and Research Center IRCCS Rozzano Italy; ^13^ Skin and Allergy Hospital Helsinki University Hospital University of Helsinki Helsinki Finland; ^14^ Division of Internal Medicine Asthma and Allergy Barlicki University Hospital Medical University of Lodz Lodz Poland; ^15^ Rhinology Unit & Smell Clinic ENT Department Hospital Clinic Clinical & Experimental Respiratory Immunoallergy, IDIBAPS, CIBERES University of Barcelona Barcelona Spain; ^16^ Department of Pharmacy CIRFF Center of Pharmacoeconomics and Drug Utilization Research University of Naples Federico II Naples Italy; ^17^ Department of Prevention of Environmental Hazards Allergology and Immunology Medical University of Warsaw Warsaw Poland; ^18^ Nova Southeastern University Fort Lauderdale Florida USA; ^19^ International Pharmaceutical Federation The Hague The Netherlands; ^20^ Ezfy Lisbon Portugal; ^21^ University Hospital of Montpellier Montpellier France; ^22^ Fraunhofer Institute for Translational Medicine and Pharmacology ITMP Allergology and Immunology Berlin Germany; ^23^ Institute of Allergology Charité – Universitätsmedizin Berlin, Corporate Member of Freie Universität Berlin and Humboldt‐Universität zu Berlin Berlin Germany; ^24^ Quality Use of Respiratory Medicine Group, Woolcock Institute of Medical Research, The University of Sydney Sydney NSW Australia

**Keywords:** allergic rhinitis, community pharmacy, pharmacist

## Abstract

**Background:**

Allergic rhinitis (AR) management requires a coordinated effort from healthcare providers and patients. Pharmacists are key members of these integrated care pathways resolving medication‐related problems, optimizing regimens, improving adherence and recommending therapies while establishing liaisons between patients and physicians.

**Methods:**

Allergic Rhinitis and its Impact on Asthma (ARIA) first published a reference document on the pharmacist's role in allergic rhinitis management in 2004. Several guidelines were developed over the past 20 years improving the care of allergic rhinitis patients through an evidence‐based, integrated care approach.

**Results:**

This ARIA/EAACI/FIP Position Paper is based on the latest ARIA in the Pharmacy guidelines and provides: (a) a structured approach to pharmacists identifying people with AR and/or allergic conjunctivitis as well as those at risk of poor disease control; (b) an evidence‐based clinical decision support tool for optimising the management of allergic rhinitis in the community pharmacy; and (c) a framework of referral to the physician.

**Conclusion:**

This document is not intended to be a mandatory standard of care but is provided as a basis for pharmacists and their staff to develop relevant local standards of care for their patients, within their local practice environment. Pharmacy care varies between countries, and the guide should be adapted to the local situation.

## 1 INTRODUCTION

- Allergic rhinitis (AR) is the most common form of non‐infectious rhinitis and is one of the most prevalent chronic diseases.^1^, ^2^, ^3^, ^4^
- Cardinal symptoms of AR include rhinorrhoea, nasal obstruction, sneezing and nasal itching. In some cases, these symptoms are spontaneously reversible, while, in others, they can be controlled by adequate treatment.^5^
- Symptoms associated with AR have a significant impact on work^6^ and school productivity, sleep^7^ and social interactions. There is an association between symptoms of AR and decreased general health‐related quality of life.^8^, ^9^
- Allergic conjunctivitis and asthma are common multimorbidities experienced by patients with AR.^10^
- Most AR patients choose to manage their condition via self‐medication with non‐prescription medicines.^11^, ^12^, ^13^ The use of these medicines for self‐medication may be appropriate or inappropriate.^14^ Community pharmacists play a critical role assisting in the management of AR and advising on the appropriate self‐medication.^15^, ^16^
- Pharmacists may also assist in the identification of patients who are inappropriately self‐medicating resulting in a suboptimal treatment of their condition. They can also, if necessary, refer these patients for medical assessment.^17^
- The Allergic Rhinitis and its Impact on Asthma (ARIA) guidelines, which were first released 20 years ago and are continually being updated with the latest evidence, provide a guide to the latest evidence‐based integrated care approach to the management of AR.^18^

## 2 STEP 1: DIFFERENTIAL DIAGNOSIS OF ALLERGIC RHINITIS IN THE COMMUNITY PHARMACY

**FIGURE 1:**
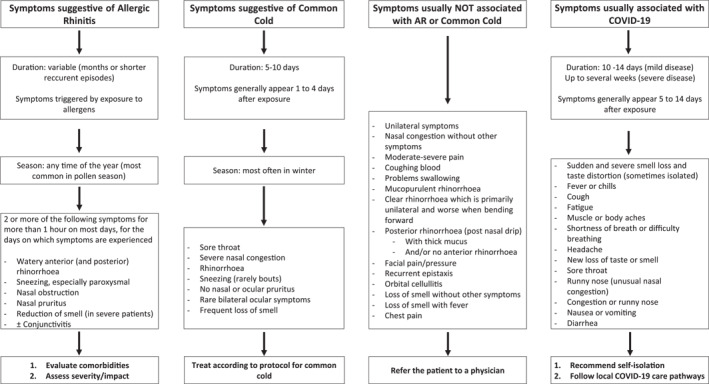
Recognising allergic rhinitis in the pharmacy.^1^ For more information on which patients should be referred to a physician (and in what time frame), see Appendix III. Please refer to local guidelines for additional symptoms for referral

- Pharmacists play an important role in confirming an AR diagnosis: some patients purchasing AR medicines in the pharmacy will have a diagnosis of AR by a physician, others will have an appropriate self‐diagnosis of AR, and the remainder no diagnosis or an incorrect one^18^ (Table 1).
- AR symptoms may be similar to those of several conditions and confused with a viral infection such as the common cold/acute rhinosinusitis (including COVID‐19) or chronic rhinosinusitis^19^, ^20^, ^21^ (Figure 1).
- The presence of **nasal itching**, **rhinorrhoea**, **sneezing** and eye symptoms is often consistent with allergic rhinitis. A mild‐to‐moderate loss of smell (hyposmia) may be present in the most severe patients, and can be sudden, severe and sometimes isolated in COVID‐19 patients^22^ (Figure 1).

**TABLE 1:** Questions to help identify allergic rhinitis

What is your main symptom? (Check for rhinorrhoea, sneezing, itchy nose, nasal congestion, loss of smell, watery or itchy eyes.)
How long have you had these symptoms?
Do you have the symptoms all the time or do they come and go?
Are you aware of anything that seems to bring the symptoms on, such as being outdoors, pollen seasons, contact with animals, something you handle at work or at home?
Has a doctor ever diagnosed hay fever, allergic rhinitis or asthma?
Is your nasal discharge clear and watery? (Purulent discharge suggests infection.)
Are you experiencing any wheezing or shortness of breath? (“Yes” may indicate asthma.)
Do you have an earache or any pain in your face? (“Yes” may indicate otitis media or rhinosinusitis.)

## 3 STEP 2a: ASSESSING COMMON AR COMORBIDITIES ‐ ALLERGIC CONJUNCTIVITIS

**FIGURE 2:**
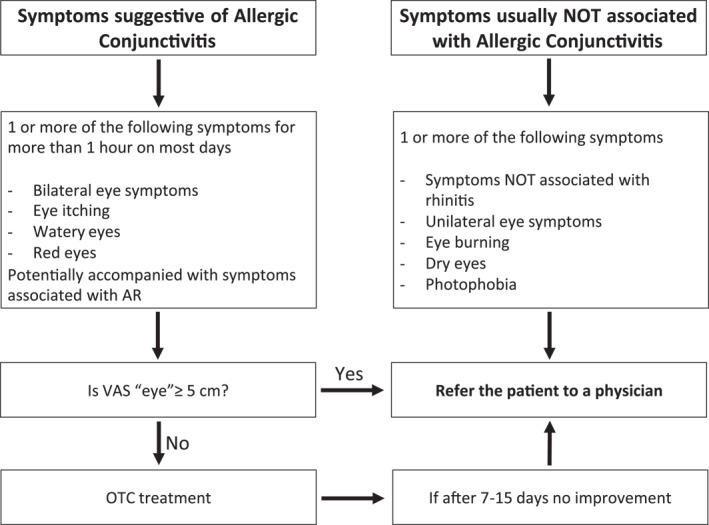
Screening of Allergic Conjunctivitis in the pharmacy^2^

- Eye symptoms are common in AR patients. However, they are not experienced by all AR patients.^23^
- The presence of conjunctivitis should always be assessed in patients with AR symptoms (Table 2 and Figure 2).
- Importantly, conjunctivitis is not always caused by an allergen (e.g. chemical, irritant, bacterial, viral)
- Photophobia (light sensitivity), eye burning, dry eyes and unilateral symptoms are unlikely to be associated with Allergic Conjunctivitis and need a physician evaluation.^24^

**TABLE 2:** Questions to help identify allergic conjunctivitis

What is your main symptom? (Check for bilateral eye symptoms, eye itching, watery eyes, red eyes.)
Do you have allergic rhinitis?
Do your eyes burn? (“Yes” may indicate disease other than allergic rhinitis.)
Do your eyes burn? (“Yes” may indicate disease other than allergic rhinitis.)
Do you have photophobia? (“Yes” may indicate disease other than allergic rhinitis and the patient should be referred to a doctor.)

## 4 STEP 2b: ASSESSING COMMON AR COMORBIDITIES ‐ ASTHMA

**FIGURE 3:**
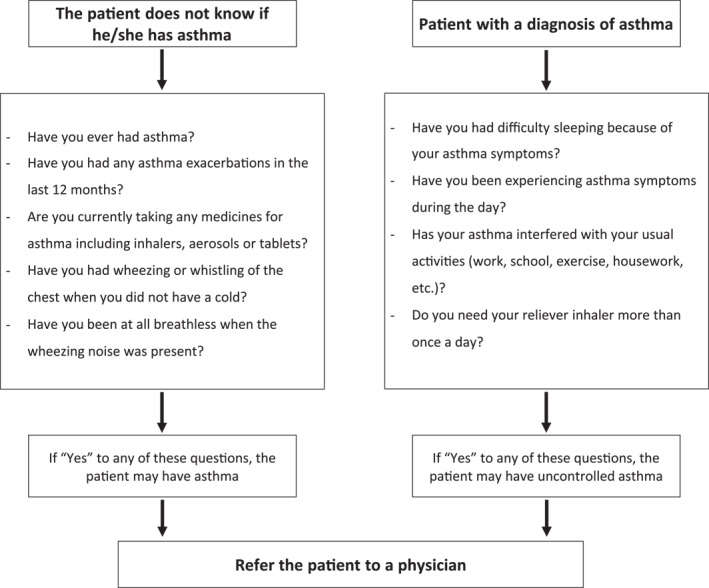
Screening of Asthma in AR patients in the pharmacy

- AR and asthma often coexist, and asthma should always be evaluated in a patient presenting with allergic rhinitis symptoms^25^, ^26^ (Figure 3).
- AR is a risk factor for the development of asthma.^27^
- In patients with asthma, AR may be associated with poor control of the disease.^25^, ^28^

## 5 STEP 3: ASSESSING THE SEVERITY OF ALLERGIC RHINITIS AND/OR ALLERGIC CONJUNCTIVITIS

The ARIA guidelines propose a classification of AR based on symptom control, quality of life, daily impact and duration. All of these can be combined into one question which relates to the degree to which the AR is bothersome^25^, ^29^:

VAS “Nose” (0–10 cm): “How much are your nose symptoms bothering you today?”
VAS “Eyes” (0–10 cm): “How much are your eye symptoms bothering you today?”

**FIGURE 4:**
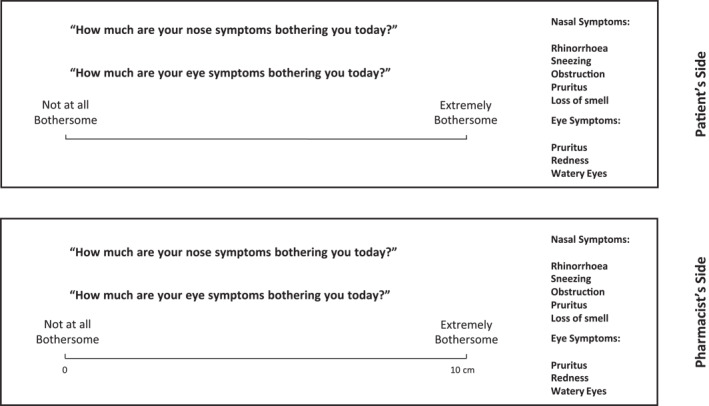
Determining the impact of Allergic Rhinitis and/or Allergic Conjunctivitis symptoms using VAS

Allergic rhinitis may be intermittent or persistent, but this does not influence the treatment to be recommended. The ARIA guidelines base treatment recommendations on the impact of symptoms on day‐to‐day living^5^, ^25^, ^30^ (Figure 4).

**FIGURE 5:**
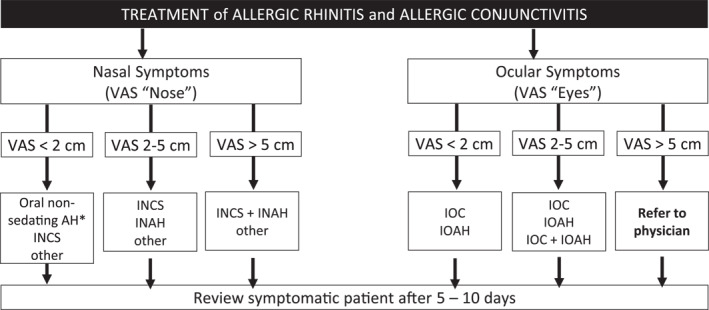
Treatment of allergic rhinitis in the pharmacy (adapted from^18^, ^31^). AH, antihistamine; INAH, intranasal antihistamine; INCS, intranasal corticosteroid; IOAH, intraocular antihistamine; IOC, intraocular cromone. *If nasal congestion is the main symptom; INCS if coexisting asthma.^32^ This algorithm should be adapted to the regulations, needs, price of medications and cultural barriers of each country or region. VAS “Nose” (0–10 cm): “How much are your nose symptoms bothering you today?” VAS “Eyes” (0–10 cm): “How much are your eye symptoms bothering you today?”

## 6 STEP 4: TREATMENT OF ALLERGIC RHINITIS IN THE PHARMACY (FIGURE 5)

By comparing AR control at the first dispensing of an OTC medication with the evolution of control during treatment, the algorithm can help both the pharmacist and the physician to optimise treatment (APPENDIX I and APPENDIX II).

The monitoring and self‐management of AR can be supported through the MASK‐air App^33^ which can be downloaded for iPhone or Android (https://www.mask‐air.com/).

The cut‐off value for VAS “eye” is based on the results of the AR and the group's opinion, but has not been validated.

## 7 STEP 5: LONG‐TERM MONITORING AND PATIENT SUPPORT

**FIGURE 6:**
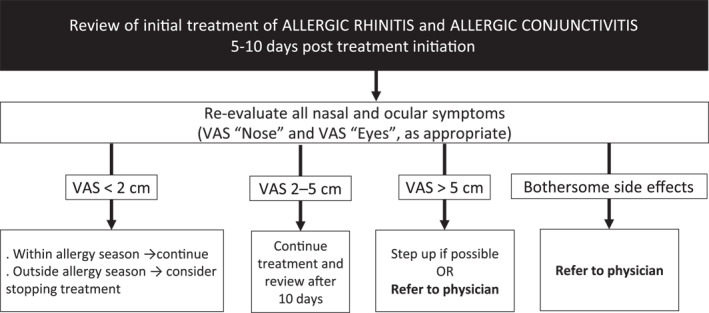
Follow‐up of AR treatment. AH, antihistamine; INAH, intranasal antihistamine; INCS, intranasal corticosteroid; IOAH, intraocular antihistamine; IOC, intraocular cromone. *If nasal congestion is the main symptom; INCS if coexisting asthma. This algorithm should be adapted to the regulations, needs, price of medications and cultural barriers of each country or region. VAS “Nose” (0–10 cm): “How much are your nose symptoms bothering you today?” VAS “Eyes” (0–10 cm): “How much are your eye symptoms bothering you today?”

It is critical that people with AR should be followed‐up over time, to ensure that their treatment is appropriate and to identify patients who require a referral to their physician (APPENDIX III). Follow‐up should occur 5–10 days post‐treatment initiation (Figure 6).^18^, ^31^

In considering how to assist the patient in long‐term management, it is important to realise that AR is undertreated and underdiagnosed and that patient self‐selection is profound.^34^ Patients often trivialise AR and do not realise the extent to which their AR presents a burden to their day‐to‐day living. Therefore, educating patients on recognising the impact of AR and counselling them on the goals they would like to achieve has been shown to be effective.^35^, ^36^ At this stage, unfortunately, there is much work to be done over time to ensure that patients remain adherent to their AR treatment^37^ (Appendix IV).

The pharmacist should continue to suggest to the patient that the monitoring and self‐management of AR can be supported through the MASK‐air App^33^ (which can be downloaded for iPhone or Android (https://www.mask‐air.com/)).

## AUTHOR CONTRIBUTIONS

Olga Lourenco: Conceptualization; Equal, Methodology; Equal, Writing – original draft; Equal, Writing – review & editing; Equal, Biljana Cvetkovski: Writing – original draft; Equal, Writing – review & editing; Equal, Vicky Kritikos: Writing – original draft; Equal, Writing – review & editing; Equal, Rachel House: Writing – original draft; Equal, Writing – review & editing; Equal, Sophie Scheire: Writing – original draft; Equal, Writing – review & editing; Equal, Elisio M Costa: Writing – original draft; Equal, Writing – review & editing; Equal, Joao Fonseca: Writing – original draft; Equal, Writing – review & editing; Equal, Enrica Menditto: Writing – review & editing; Equal, Anna Bedbrook: Writing – review & editing; Equal, Slawomir Bialek: Writing – review & editing; Equal, Vitalis Briedis: Writing – review & editing; Equal, Koen Boussery: Writing – review & editing; Equal, Giorgio Walter Canonica: Writing – review & editing; Equal, Tari Haahtela: Writing – review & editing; Equal, Piotr Kuna: Writing – review & editing; Equal, Joaquim Mullol: Writing – review & editing; Equal, Valentina Orlando: Writing – review & editing; Equal, Bolesław Samoliński: Writing – review & editing; Equal, Dana Wallace: Writing – review & editing; Equal, Catherine Duggan: Writing – review & editing; Equal, Ema Paulino: Writing – review & editing; Equal, Goncalo Sousa Pinto: Writing – review & editing; Equal, Lars‐Ake Soderlund: Writing – review & editing; Equal, Jean Bousquet: Conceptualization; Equal, Supervision; Equal, Writing – review & editing; Equal, Sinthia Bosnic‐Anticevich: Conceptualization; Equal, Methodology; Equal, Supervision; Equal, Writing – original draft; Equal, Writing – review & editing; Equal.

## CONFLICT OF INTEREST

Sinthia Bosnic‐Anticevich reports grants from TEVA, and personal fees from TEVA, AstraZeneca, Boehringer Ingelheim, GSK, Sanofi, and Mylan. João A. Fonseca reports personal fees from Viatris/Mylan. Piotr Kuna reports personal fees from Adamed, Berlin Chernie Menarini, Boehringer Ingelheim, Chiesi, AstraZeneca, Krka, Angellini, Novartis, Polpharma, Lekam, and GSK. Ema Paulino is the President of the Portuguese National Pharmacy Association and has been a Board member of the International Pharmaceutical Federation until October 2021. Biljana Svetkovski reports personal fees from GSK and Sanofi. Dana Wallace reports personal fees from Optinose, ALK, and Sanofi, and was a primary author on the JTFPP Rhinitis Practice Parameter Update 2020.

## Supporting information

Supplementary Material

## ENDNOTES

^1^
In Figure 1, some of the items not associated with AR symptoms refer to rhinosinusitis and other diseases that need to be checked during differential diagnosis.

^2^
The cut‐off value for VAS “eye” is based on the results of the AR and on the group's opinion, but has not been validated. For determining VAS “eye”, see Figure 4.
