# Supplementary material for: Management of allergic rhinitis symptoms in the pharmacy Pocket guide 2022
Source: Clin Transl Allergy. 2022 Oct 5;12(10):e12183. doi: 10.1002/clt2.12183 (PMC9533218; doi:10.1002/clt2.12183)
Supplement: Supplementary file 1 — Supplementary Material [file CLT2-12-e12183-s001.docx]

**APPENDIX I** – Allergic Rhinitis Decision Support Tool.


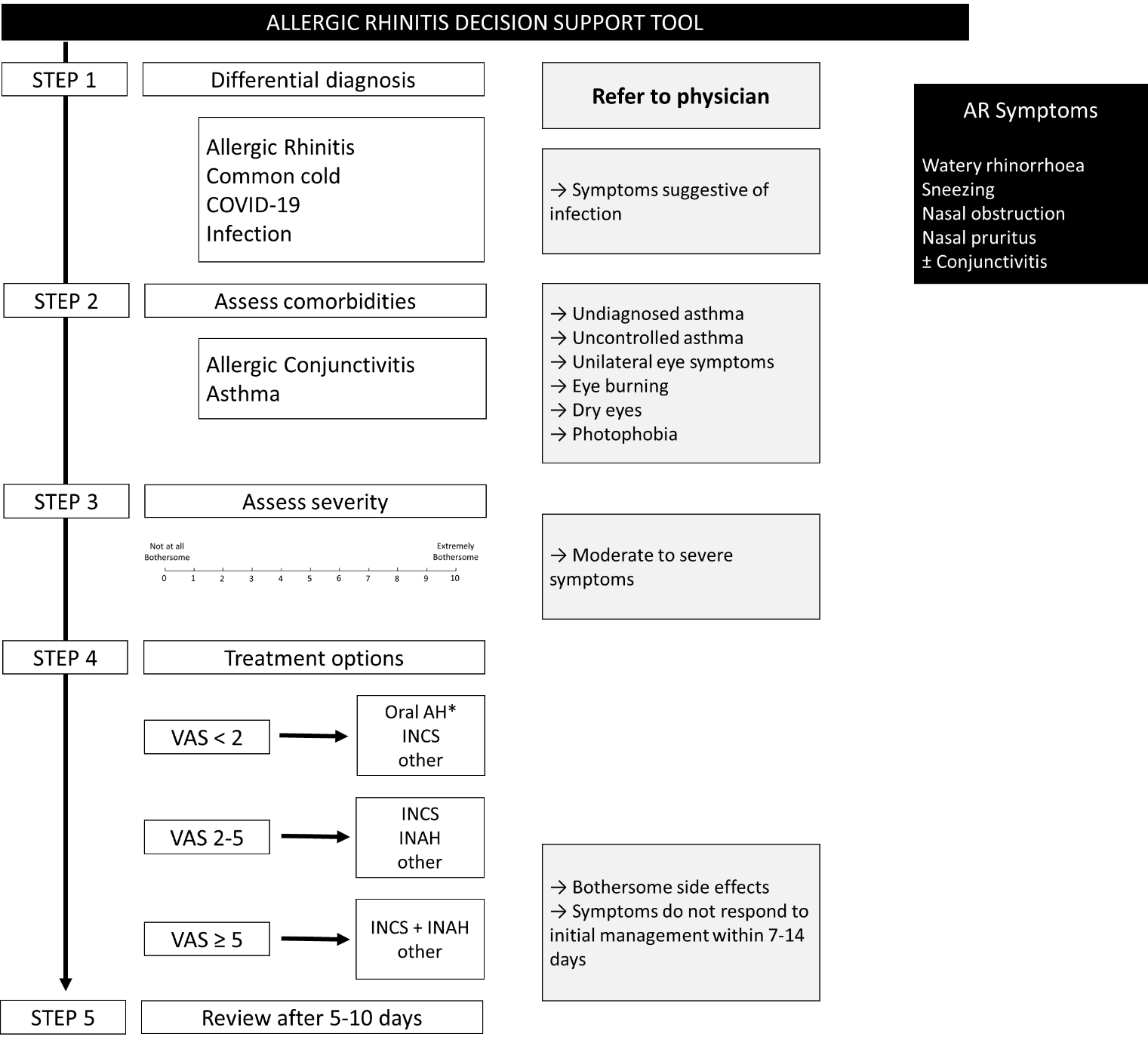


**APPENDIX II** – Common medications available for the treatment of allergic rhinitis (including prescription-only medicines).

| **Oral Antihistamines (2^nd^ generation)** | **Intranasal Antihistamines** | **Ocular Antihistamines** |
| --- | --- | --- |
| Acrivastine  Azelastine  Bilastine  Cetirizine  Desloratadine  Ebastine  Fexofenadine  Levocetirizine  Loratadine  Mizolastine  Rupatadine | Azelastine  Levocabastine  Olopatadine | Alcaftadine  Azelastine  Bepotastine  Ketotifen  Emedastine  Epinastine  Olopatadine |
| **Oral corticosteroids** | **Intranasal corticosteroids** |  |
| Betamethasone  Deflazacort  Dexamethasone  Hydrocortisone  Methylprednisolone  Prednisolone  Prednisone  Triamcinolone | Beclomethasone  Budesonide  Ciclesonide  Fluticasone propionate  Fluticasone furoate  Flunisolide  Mometasone  Triamcinolone |  |
|  | **Intranasal corticosteroids + antihistamine formulations** |  |
|  | Azelastine + Fluticasone  (MP-AzeFlu)  Olopatadine + Mometasone |  |
|  | **Intranasal Chromones** | **Intraocular Chromones** |
|  | Sodium cromoglycate  Nedocromil | Sodium cromoglycate  Nedocromil |
|  |  | **Ocular Mast cell Stabilizers** |
|  |  | Pemirolast  Lodoxamide |
| **Oral decongestants** | **Intranasal decongestants** |  |
| Ephedrine  Phenylephrine  Phenylpropanolamine  Pseudoephedrine | Ephedrine  Naphazoline  Oxymetazoline  Phenylephrine  Tetrahydrozoline  Tramazoline  Xylometazoline |  |
| **Oral Anti-leukotrienes** |  |  |
| Montelukast  Pranlukast  Zafirlukast  Zileuton |  |  |

**APPENDIX III** – Summary of reasons for referral to a physician.

| **Referral to a physician should be considered in cases where:** |
| --- |
| - Moderate to severe symptoms of AR are present, although initial treatment might be provided by a pharmacist while waiting to see a physician; - There are symptoms suggestive of undiagnosed asthma or uncontrolled asthma in patients with a previous diagnosis of asthma; - There are symptoms suggestive of infection; - Symptoms do not respond to initial management within 7-14 days; - Bothersome side effects are experienced; - The patient is a child of less than 12 years of age, is a pregnant woman, takes multiple medications, or has a complex multimorbidity. |

**APPENDIX IV** – Self-management strategies for the patient.

| **If you have allergic rhinitis:** |
| --- |
| - Avoid allergen triggers whenever possible; - If you have a pollen allergy, check the pollen forecast for your area; pollen counts are at their highest early in the morning, in the evening and at night; avoiding grassy open spaces at these times may be helpful; - If you have a dust mite allergy, reduce dust and dust mites in the home; - If you have a confirmed animal allergy, avoid animals and surfaces likely to be contaminated with fur and dander; - Use a symptoms diary (paper, online, app); - Good technique is important for intranasal medications, refer to your community pharmacist and manufacturer instructions if you forget how to use them correctly; - Set reminders to medication times; - If your symptoms remain uncontrolled after 7 days of using your medication, contact your community pharmacist or attending physician; - Antibiotics are not effective in the treatment of allergic rhinitis; - Contact your community pharmacist or attending physician if troublesome symptoms arise; - In case of any doubts, contact your community pharmacist; - Remember, allergic rhinitis is a chronic condition, but it can be optimally managed. |
